# Receptor-targeted therapy of human experimental urinary bladder cancers with cytotoxic LH-RH analog AN-152 (AEZS-108)

**DOI:** 10.18632/oncotarget.546

**Published:** 2012-07-22

**Authors:** Karoly Szepeshazi, Andrew V. Schally, Gunhild Keller, Norman L. Block, Daniel Benten, Gabor Halmos, Luca Szalontay, Irving Vidaurre, Miklos Jaszberenyi, Ferenc G. Rick

**Affiliations:** ^1^ Veterans Affairs Medical Center Miami, FL; ^2^ South Florida VA Foundation for Research and Education, Miami, FL; ^3^ Department of Pathology, University of Miami, Miller School of Medicine, Miami, FL; ^4^ Division of Hematology/Oncology University of Miami, Miller School of Medicine, Miami, FL; ^5^ Division of Endocrinology, Department of Medicine, University of Miami, Miller School of Medicine, Miami, FL; ^6^ Section of Hematology/Oncology, University Clinic, Hamburg, Germany; ^7^ Department of Gastroenterology University Clinic, Hamburg, Germany; ^8^ Department of Biopharmacy, School of Pharmacy, University of Debrecen, Hungary

**Keywords:** urinary bladder, urothelial cancer, targeted therapy, LH-RH receptor, cytotoxic, doxorubicin

## Abstract

Many bladder cancers progress to invasion with poor prognosis; new therapeutic methods are needed. We developed a cytotoxic LH-RH analog, AN-152 (AEZS-108) containing doxorubicin (DOX), for targeted therapy of cancers expressing LHRH receptors. We investigated the expression of LH-RH receptors in clinical bladder cancers and in HT-1376, J82, RT-4 and HT-1197 human bladder cancer lines. The effect of analog, AN-152, on growth of these tumor lines xenografted into nude mice was analyzed. Using molecular and functional assays, we also evaluated the differences between the effects of AN-152, and DOX alone. We demonstrated the expression of LH-RH receptors on 18 clinical bladder cancers by immunohistochemistry and on four human urinary bladder cancer lines HT-1376, J82, RT-4 and HT-1197 by Western blotting and binding assays. AN-152 powerfully inhibited growth of these bladder cancers in nude mice. AN-152 exerted greater effects than DOX and was less toxic. DOX activated strong multidrug resistance mechanisms in RT-4 and HT-1197 cancers, while AN-152 had no or less such effect. PCR assays and *in vitro* studies revealed differences in the action of AN-152 and DOX on the expression of genes involved in apoptosis. These results suggest that targeted cytotoxic LH-RH analog, AN-152 (AEZS-108), should be examined for treatment of patients with LH-RH receptor positive invasive bladder cancers.

## INTRODUCTION

Yearly almost 400,000 new cases of urinary bladder cancer are diagnosed in the world and more than 150,000 people die of the disease [1]. In the US, bladder cancer is the fifth most frequent malignancy and the most expensive tumor to treat [2]. Approximately 75% of bladder cancers are diagnosed at an early stage; half of these progress to invasive tumor [2].

Chemotherapy for the treatment of metastatic or recurrent transitional cell carcinoma of the urinary tract began decades ago. Doxorubicin (DOX) and cisplatin as single agents or in various combinations showed efficacy in the treatment of advanced bladder cancer and, in combinations with cyclophosphamide, methotrexate, vinblastine, reached response rates of 90% [3, 4]. The combination of methotrexate, vinblastine, adriamycin and cisplatin (M-VAC) increased median survival of patients up to 12.5 months [5]. These response rates were accompanied by significant systemic toxicity and frequent relapse due to resistance to additional therapy [5-7]. Combinations of gemcitabine and cisplatin show response rates similar to the M-VAC [8] with less severe side effects and are considered a standard of care for patients with metastatic disease [9].

Elucidation of the molecular characteristics of urothelial cancers introduced possibilities for targeted therapies. Receptors for growth factors appear to play a role in progression of urothelial carcinoma [10]. Thus, targeting receptor tyrosine kinases, e.g. EGF, Her-2/neu, or manipulation of signal transduction pathways provide new therapeutic strategies [11, 12] but require molecular analyses to select patients who would benefit [13].

The demonstration of receptors for neuropeptide hormones on various tumor cells [14-16] led to development of cytotoxic peptides and peptide hormones linked to radionuclides for tumor diagnosis and therapy [17-19]. Radiolabeled analogs of somatostatin, bombesin or vasoactive intestinal peptide (VIP) are now increasingly used for tumor imaging and therapy [17-19]. Our group synthesized analogs of LH-RH, somatostatin, and bombesin linked to DOX which selectively target tumors expressing the specific receptors while sparing normal tissues from toxicity. These analogs inhibit growth of various experimental human cancers, and are more effective and tolerable than the cytotoxic radical, DOX, alone [17-19].

Initially, receptors for LH-RH were demonstrated on human breast, endometrial, ovarian, and prostatic cancers [17-20]. Subsequently expression of these receptors was also found on human non-Hodgkin's lymphomas [21], renal cell carcinomas [22] and malignant melanomas [23], suggesting the potential of targeted therapy with cytotoxic analogs of LH-RH. Cytotoxic LH-RH analog, AN-152 (AEZS-108), showed promising results in phase II clinical trials in women with several gynecological cancers [24, 25] and is now in phase I/II clinical trials for patients with prostate [26] and bladder cancers [19, 27]. In this study we investigated the expression of LH-RH receptors in clinical human urinary bladder specimens and in four human bladder cancer cell lines. We also analyzed the effect of cytotoxic LH-RH analog, AN-152 (AEZS-108), on growth of human experimental tumors xenografted into nude mice. We compared the effect of AN-152 and its cytotoxic radical, DOX, by molecular and functional assays.

## RESULTS

### LH-RH receptor expression in human bladder cancer

**Figure 1:**
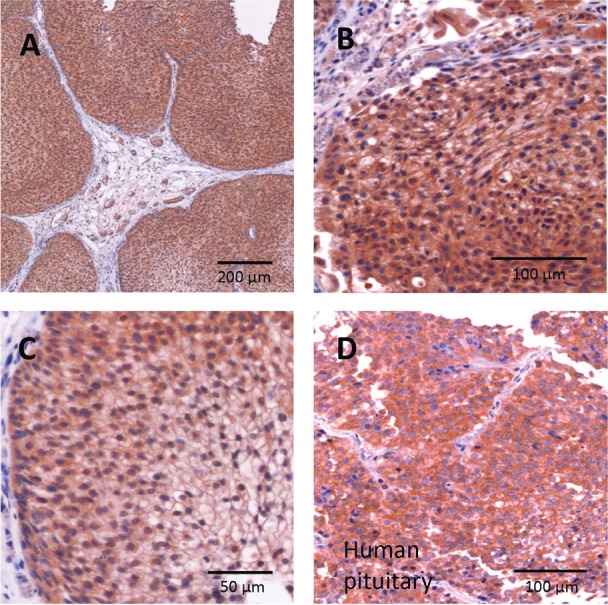
Expression of LH-RH receptors in human bladder carcinomas The tissue was stained by immunohistochemistry with primary LH-RH receptor antibody. A-C: urothelial bladder carcinoma; D: positive control, anterior pituitary.

Eighteen human primary urothelial cancer samples were evaluated by immunohistochemistry. Positive staining for LH-RH receptors was observed in all specimens (Fig. 1 a-c). Enhanced staining of the plasma membrane as well as cytoplasmic staining were detected in malignant cells and the positive control (human anterior pituitary) (Fig. 1d). In three samples, high levels of LH-RH receptor expression with more than 75% positively stained malignant cells was found, two samples revealed weak expression; 13 samples were intermediate. Expression was variable; areas of high, distinct and low LH-RH receptor density were sometimes seen within one sample. In these cases, the dominating receptor density was chosen for final categorization. In surrounding non-malignant tissue no or marginal LH-RH receptor expression was found (Fig. 1a-c).

### Effect of treatments on tumor growth in nude mice

**Figure 2:**
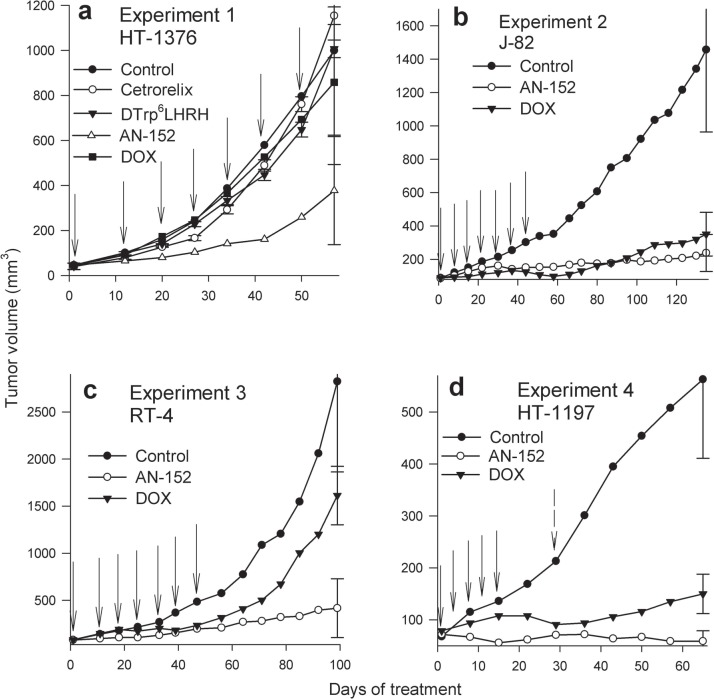
Effect of treatment with cytotoxic LH-RH analog, AN-152 (AEZS-108), and doxorubicin (DOX), on growth of human bladder cancers in nude mice The vertical bars represent SE. Solid arrows show treatments with both cytotoxic compounds, dashed arrow shows treatment only with AN-152 (2d).

In Experiment 1, AN-152 strongly inhibited HT-1376 cancers. Tumor volume and tumor weights were significantly less than control (Fig. 2a) (Table 1). In contrast, DOX, Cetrorelix and [D-Trp^6^]LH-RH had no effect on tumor growth. The median growth rate values of the tumors treated with AN-152 and DOX differed significantly (P=0.018). Body and organ weights were similar in all groups, except for ovarian weights, which were lower in the AN-152 group (also in Experiments 3 and 4) (data not shown).

**Table 1:** Growth characteristics of human urinary bladder cancers in nude mice and changes in body weights of the animals after treatment with LH-RH analogs AN-152 or DOX

Groups	Tumor volume (mm3)	Tumor weights (mg)	Body weights (g)
Experiment 1	HT-1376		
1. Control	999 ± 375	900 ± 340	27.3 ± 1.5
2. Cetrorelix	1154 ± 438	623 ± 124	28.6 ± 0.6
3. [D-Trp^6^]LH-RH	1007 ± 349	648 ± 161	24.6 ± 0.6
4. AN-152	377 ± 240	256 ± 139*	26.2 ± 0.9
5. DOX	858 ± 365	396 ± 96	24.2 ± 0.6*
Experiment 2	J82		
1. Control	1456 ± 493	1256 ± 384	29.2 ± 0.9
2. AN-152	238 ± 111*	360 ± 181	24.3 ± 0.9*
3. DOX	351 ± 131	386 ± 181	21.6 ± 0.6*†
Experiment 3	RT-4		
1. Control	2824 ± 961	1353 ± 532	24.6 ± 0.6
2. AN-152	416 ± 313*	475 ± 339*	24.3 ± 1.1
3. DOX	1613 ± 764	868 ± 673	22.0 ± 1.6*
Experiment 4	HT-1197		
1. Control	563 ± 152	527 ± 173	28.9 ± 1.5
2. AN-152	60 ±20*	54 ± 30*	25.3 ± 0.9
3. DOX	150 ± 38*†	70 ± 23	23.1 ± 1.1*

Values are means ± SE.

* P<0.05 vs. Control

† P<0.05 vs. AN-152 group

In Experiment 2 (Fig. 2b), both AN-152 and DOX at first powerfully inhibited growth of J82 tumors. The tumors treated with DOX started regrowing at day 80 while those treated with AN-152 continued inhibition. At the end of the experiment, AN-152 produced a significant (84%) reduction in volume. The DOX decrease was less (76%) and not significantly different from control. Tumor weights and body weights were lower than control in both treated groups (Table 1). The mice that received DOX were emaciated; their mean weight was significantly lower than that of controls or animals treated with AN-152 (Table 1).

In Experiment 3, AN-152 strongly inhibited growth of RT-4; the tumors did not resume growth after treatment cessation (Fig. 2c). DOX initially reduced RT-4 proliferation, the tumors started growing more intensely after treatment cessation. Tumor weights were also lower after treatment with AN-152, but not DOX (Table 1). DOX again significantly lowered animal body weights (Table 1).

In Experiment 4, both AN-152 and DOX produced a substantial volume reduction of HT-1197 cancers; the effect of AN-152 was significantly greater than that of DOX (Fig. 2d). Tumor weights were lower only in the group receiving AN-152 (Table 1). DOX significantly reduced the weights of mice at day 29, these remained significantly lower to the end of the experiment (Table 1). Thus, Group 2 received another AN-152 injection on day 30, but DOX was discontinued because of systemic toxicity.

### Receptor assays and Western blots

**Figure 3:**
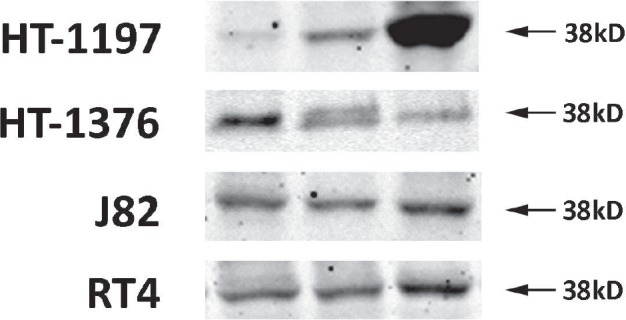
Detection LH-RH receptor protein (38 KD) by Western blotting All four human urinary bladder cancer lines grown in nude mice expressed LH-RH receptors. Representative blots of three independent experiments are shown.

Radiolabeled [D-Trp^6^]LH-RH was bound to a single class of specific binding sites on all four cancer lines. The concentrations of LH-RH receptors and the binding affinity varied slightly among the tumor models, as shown in supplementary Table S1. LH-RH receptor protein (38 KD) was detected in all four tumors by Western blotting, the levels of receptor protein being not significantly different between treated group and control (Fig. 3).

### Molecular analysis

**Figure 4:**
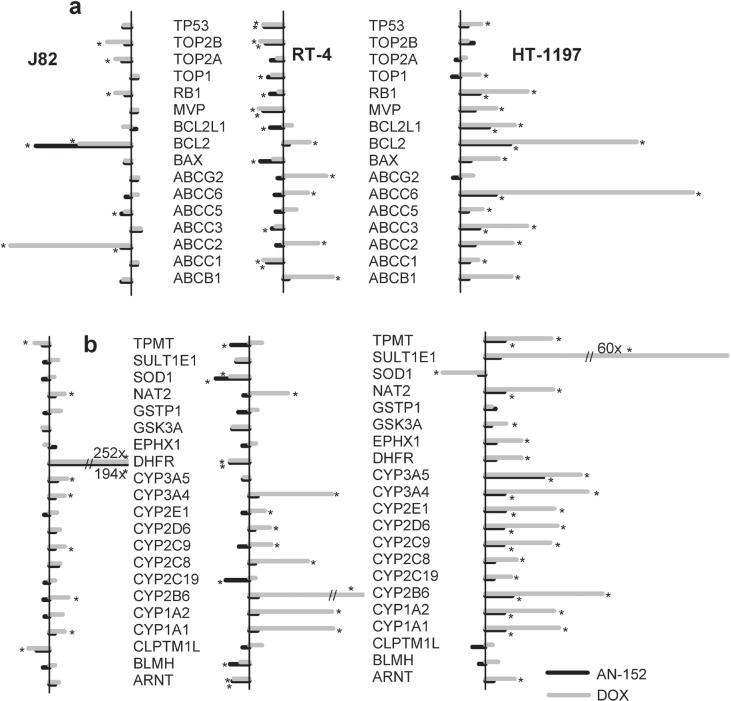

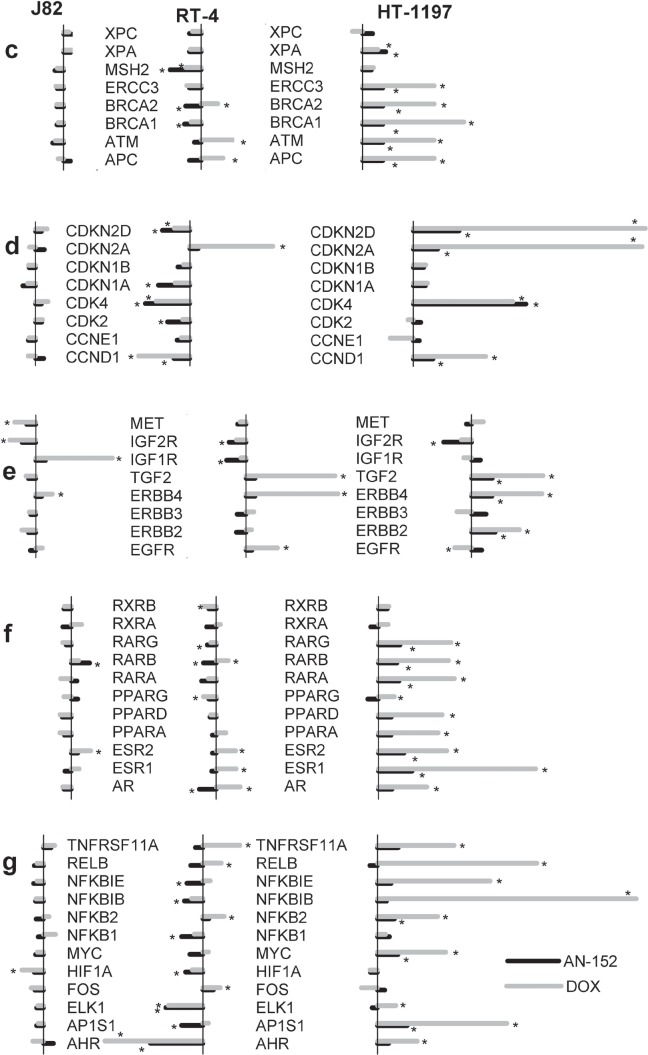
Human urinary bladder cancers grown in nude mice were analyzed with the Human Cancer Drug Resistance & Metabolism RT Profiler PCR Array The vertical bars on the left shows 10-fold change compared to control *= P<0.05 vs. control. **(A)Changes in genes involved in drug resistance.** ABCB1: ATP-binding cassette, subfamily B, member 1; ABCC1-6: ATP-binding cassette, subfamily C, members 1-6; ABCG2: ATP-binding cassette, subfamily G, member 2; BAX: BCL2-assiciated X protein; BCL2: B-cell CCL/lymphoma 2; BCL2L1: BCL2-like 1; MVP: Major vault protein; RB1: Retinoblastoma 1; TOP1: Topoisomerase (DNA) I; TOP2A: Topoisomerase (DNA) II alpha; TOP2B: Topoisomerase (DNA) II beta; TP53: Tumor protein p53. **(B)Changes in genes involved in drug metabolism.** ARNT: Aryl hydrocarbon receptor nuclear translocator; BLMH: Bleomycin hydrolase; CLPTM1L: Cleft lip and palate transmembrane protein 1-like protein (cisplatin resistance-related protein); CYP1A1, CYP1A2, CYP2B6, CYP2C19, CYP2C8, CYP2C9, CYP2D6, CYP2E1, CYP3A4, CYP3A5: Cytochrome P450, family 1-3, subfamily A-D, polypeptide 1-19; DHFR: Dihydrofolate reductase; EPHX1: Epoxide hydrolase, microsomal (xenobiotic); GSK3A: Glycogen synthase kinase 3 alpha; GSTP1: Glutathione S-transferase pi 1; NAT2: N-acetyltransferase 2; SOD1: Superoxide dismutase 1; SULT1E1: Sulfotransferase family 1E, estrogen-preferring, member 1; TPMT: Thiopurine S-methyltrasferase. **(C)Changes in genes involved in DNA repair.** *1*: APC: Adenomatous polyposis coli; ATM: Ataxia teleangiectasia mutated; BRCA1, BRCA2: Breast cancer 1, 2; ERCC3: Excision repair cross-complementing rodent repair deficiency, complementation group 3 (xeroderma pigmentosum group B complementing); MSH2:MutS homolog 2, colon cancer, nonpopyposis type 1; XPA, XPC: Xeroderma pigmentosum, complementation group A, C. **(D)Genes involved in cell cycle.** CCND1, CCNE1: Cyclin D1, E1; CDK2, CDK4: Cyclin dependent kinase 2, 4; CDKN1A, CDKN1B, CDKN2A: CDKN2D: Cyclin-dependent kinase inhibitor 1A, 1B, 2A, 2D. **(E)Changes in growth factor genes.** EGFR: Epidermal growth factor receptor; ERBB2, ERBB3, ERBB4: V-erb-b2 erythroblastic leukemia viral oncogene homolog 2, 3, 4:FGF2: Fibroblast growth factor 2; IGF1R, IGF2R: Insulin-like growth factor 1, 2 receptor; MET: Met proto-oncogene (hepatocyte growth factor receptor). **(F)Hormone receptor genes.** AR: Androgen receptor;ESR1, ESR2: Estrogen receptor 1, 2; PPARA, PPARD, PPARG: Peroxisome proliferator-activated receptor alpha, beta, gamma; RARA, RARB, RARG: Retioic acid receptor alpha, beta, gamma; RXRA, RXRB: Retinoid X receptor alpha, beta. **(G)Genes related to transcription factors.** AHR: Aryl hydrocarbon receptor; AP1S1: Adaptor-related protein complex1, sigma 1 subunit; ELK1: ELK1, member of ETS oncogene family; FOS: FBJ murine osteosarcoma viral oncogene homolog; HIF1A: Hypoxia inducible factor 1, alpha subunit; MYC: V-myc myelocytomatosis viral oncogene homolog; NFKB1, NFKB2, NFKBIB, NFKBIE: Nuclear factor of kappa light polypeptide gene enhancer in B cells1, 2, inhibitor beta, epsilon; RELB: V-rel reticuloendotheliosis viral oncogene homolog B; TNFRSF11A: Tumor necrosis factor receptor superfamily, member 11a.

Using the Cancer Drug Resistance and Metabolism PCR Array, we analyzed the expression of 84 genes that may influence response to chemotherapy. The assays involve genes related to drug resistance, drug metabolism, DNA repair, transcription, and cell cycle regulation, as well as those encoding receptors for growth factors and hormones. Three bladder cancer lines (J82, RT-4 and HT-1197) were investigated and the results are presented in Figures 3-4. The degree and pattern of changes in gene expression were different in each of the three. J82 showing the least and HT-1197 the strongest, alterations. In J82 tumor, genes related to drug resistance were not changed, except ABCC2 (ATP-binding cassette, sub-family B, member 1), which was reduced by DOX. In RT-4 cancers, DOX produced a moderate increase of 5 and a minor decrease of 4 genes, while AN-152 caused a slight decrease in 6. Practically all drug resistance genes were overexpressed in HT-1197 tumors treated with either agent; the increase was much greater after DOX (Fig. 4a). Nearly all genes involved in drug metabolism were increased by DOX in all tumors, while treatment with AN-152 caused only slight amplifications of these genes in HT-1197. (Fig. 4b). Genes engaged in DNA repair were not affected by either treatment in J82, moderately changed in RT-4 and strongly increased in HT-1197 tumors; DOX therapy induced stronger alterations (Fig. 4c). Fig. 4d demonstrates that genes encoding cyclines and cycline dependent kinases were similarly changed by the two compounds, while DOX caused a stronger increase in kinase inhibitors. Regarding growth factors and their receptor genes, DOX increased EGF receptor (EGFR), ErbB2, ErbB4 (V-erb-b2 erythroblastic leukemia viral oncogene homolog 2 and 4), fibroblast growth factor 2 FGF2 and IGF-1 receptor (IGF-1R) in various tumors, while AN-152 therapy resulted in small increases in ErbB2, ErbB4 and FGF2 in HT-1197 cancers only (Fig. 4e). Increased expression in a variety of hormone receptor genes was the strongest in HT-1197 tumors, with DOX having a much greater effect than AN-152 (Fig. 4f). Genes related to transcription factors were mostly down-regulated in RT-4 and up-regulated in HT-1197 cancers; DOX had a more powerful effect in the latter (Fig. 4g).

We also used the Human Apoptosis PCR Array to detect changes in expression of 84 genes involved in programmed cell death. Anti-apoptotic gene expression was increased by DOX in all three tumors, affecting a few genes only in J82, more in RT-4 and the most in HT-1197. AN-152 resulted in a moderate increase in anti-apoptosis genes in HT-1197 cancers (Supplementary Fig. S1a). Regarding pro-apoptotic genes, DOX produced stronger increases than AN-152 in RT-4 tumors (Supplementary Fig. S1b).

Protein analysis by Western blot showed increases in anti-apoptotic proteins, Bcl2 and BclX, in all treated tumors (results not shown), more in those treated with DOX. The differences were statistically not significant.

### Multi-drug resistance and apoptosis assays *in vitro*

**Figure 5:**
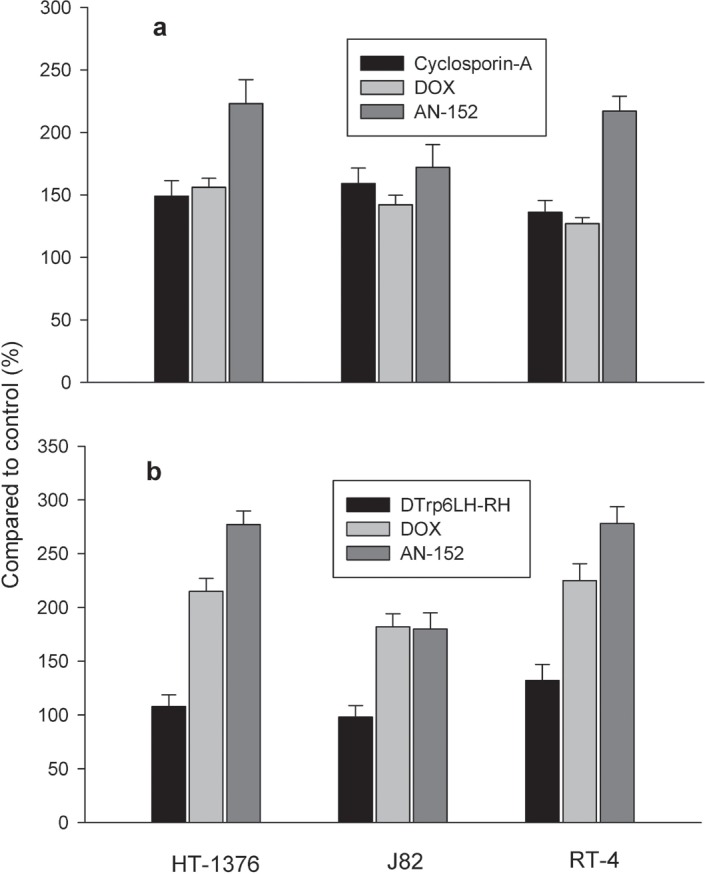
Effects of AN-152, and DOX on HT-1376, J82 and RT-4 human urinary bladder cancer cells in vitro 5a: calcein retention in the cells analyzed with MDR Assay Kit and 5b: apoptosis investigated with the Multi-Parameter Apoptosis Assay.

The MDR study showed that treatment with either cytotoxic compound resulted in a retention of calcein in all three tumor cell lines, but the retention was significantly higher after treatment with AN-152 than DOX. The greatest differences between the effect of AN-152 and DOX were in RT-4 and HT-1376 and the least in J82 (Fig. 5a). Apoptosis assay *in vitro* revealed that AN-152 had a stronger apoptogenic effect than DOX on RT-4 and HT-1376 tumor cells, while both compounds acted similarly on J82 cells (Fig. 5b).

## DISCUSSION

Siegel et al [28] estimate that 73,510 new cases of urinary bladder cancer will be diagnosed in the US in 2012 with 14,880 estimated deaths. The numbers of newly diagnosed cases and deaths are high and require improvement [28]. The estimated 5-year cost of care to Medicare is approximately one billion dollars [29].

Platinum based regimens are currently the standard of therapy but metastatic urothelial cancer remains a deadly and costly disease [5, 29, 30]. More research with novel, targeted agents is needed to improve outcomes [29].

Targeted therapies are being widely investigated and increasingly used for treatment of various tumors. Targeting produces an improvement in tumor effect and diminishes systemic toxicity [17-19, 27]. Peptide hormone receptors present on various cancer cells, can function as targets for specific compounds composed of cytotoxic agents conjugated to peptide analogs. The peptide hormone serves as a carrier molecule for homing the cytotoxic agent to target cells containing specific receptors. Thus, we have developed cytotoxic compounds containing DOX conjugated to an LH-RH agonist, tested them on a variety of experimental tumor models, and showed that they are more effective and less toxic than unconjugated DOX [17-19, 25, 27]. Besides the pituitary, receptors for LH-RH have been detected in various human cancer cell lines and human cancer specimens. These include prostate, breast, ovarian and endometrial cancers and other cancers, which are outside of the reproductive system, such as renal cell carcinoma, melanoma, Hodgkin's lymphoma and colorectal cancers [18, 19]. The expression of peptide hormone receptors on bladder cancers has been minimally investigated. Only one group verified the expression of LH-RH receptors in human bladder epithelium, bladder cancers and bladder cancer cell lines, but did not detect any effect of LH-RH on bladder cancer cells *in vitro* [31].

This study reveals the presence of LH-RH receptors (LH-RH R) in all 18 specimens of bladder cancer patients. We also demonstrated LH-RH R expression by molecular methods and binding assays of LH-RH receptors in all four human bladder cancer lines investigated. The four cell lines used are transitional cell carcinomas originating from bladder, but with various degrees of differentiation and differing in characteristics and behavior. Thus, HT-1376 originated from a grade 3 carcinoma with a functional loss mutation in p53; RT-4 is a transitional cell papilloma with wild-type p53. Clinical relevance of various tumor cell lines is linked to the clinical tumor behavior [32]. RT-4 cancer is associated with long survival, low grade, and low invasiveness. In contrast, J82 corresponds to high grade and invasion; HT-1376 has the shortest survival. The diversity of the four tumor lines investigated makes the study more clinically relevant. These cell lines also have varying sensitivity to DOX, as treatment with DOX inhibited growth of J82 and HT-1197 tumors, but not HT-1376 and RT-4 cancers. The four tumors showed consistent inhibition in response to AN-152, which had a stronger effect and was less toxic than DOX.

To damage tumor cells, anti-cancer drugs must enter the cell through the cell membrane, and remain for the time necessary for their action, avoiding several defensive mechanisms. Resistance affects many unrelated drugs and is therefore called multidrug resistance [33-35]. Some cancers are intrinsically resistant to specific drugs, others initially respond, but develop resistance during treatment. Drug resistant cells may overgrow during therapy and secondary genetic changes induced by a drug can lead to increased therapeutic resistance [34]. One mechanism in tumor cells is the drug-efflux system that consists of various molecules belonging to the ABC transporter family, and which can eject drugs such as DOX from the cells. ABC transporters include P-glycoproteins, (MDR1, multi drug resistance protein 1; ABCB1, ATP-binding cassette sub-family B, member 1), MRP1 (multi-drug resistance associated protein 1) and other related compounds [33, 35]. Gene expression analysis can elucidate specific resistance pathways [36]; identifying differences to drugs such as DOX, cisplatinum, or paclitaxel [37].

Most bladder carcinomas are initially sensitive to chemotherapy, but the majority develop resistance [38]. Tada et al [6] clearly demonstrated that, after relapse, the response rate to therapy inversely correlates with the expression of genes related to multidrug resistance (MDR1, MRP1, MRP3). Thus, a novel approach has been developed for targeting resistance related molecules in tumor cells [39, 40].

In this study, the results of the Cancer Drug Resistance and metabolism PCR Array revealed important differences between the effects of AN-152 (AEZS-108) and DOX on MDR related genes. Chronic treatment with DOX resulted in overexpression of most genes involved in drug efflux and drug metabolism in RT-4 and HT-1197 cancers, while AN-152 (AEZS-108) caused reductions or smaller increases in these genes. The gene alterations seem to persist after therapy cessation. The *in vitro* functional MDR assay also clearly showed differences among the bladder cancer cell lines in their reaction to a single treatment with DOX or AN-152 (AEZS-108). Calcein retention was significantly higher in all cell lines after treatment with AN-152, compared to that caused by DOX. These differences between the two compounds may be explained by an increased uptake by the cells of DOX incorporated in the molecule of AN-152 compared to unconjugated DOX, and also by a likely decreased transporter activity in the presence of AN-152 compared to DOX.

Many enzymes contribute to this intracellular metabolism and inactivation of cytotoxic agents; these enzymes can be induced or activated by the specific drugs. Our investigation revealed important differences between the effects of DOX and AN-152 on expression of genes related to drug metabolism. A similar pattern was observed in DNA repair genes which have important roles in responses to therapy. Thus, treatment with platinum salts is more effective on tumors which express low levels of DNA repair related genes. In the present study, DOX caused greater changes than AN-152 in DNA repair related genes making tumors less sensitive to therapy. DOX resistance can be also associated with changes in proteins involved in cell cycle regulation [41]. In our study, cell cycle associated genes in three tumor lines were variously affected by the treatments.

Regarding growth factor receptor genes, treatment with DOX increased some of these genes, such as ErbB2, ErbB4 and FGF2 in the tumors, which are up-regulated in bladder cancers and responsible for disease progression [42-44]. Moreover, ErbB4 may play a role in DOX-induced myocardial DNA damage [45]. Our results showing that DOX causes a greater increase in ErbB4 than AN-152 may explain the lack of cardiotoxicity so far seen in preclinical and clinical studies with AEZS-108 [24, 25, 27].

Most hormone receptor related genes analyzed were increased by DOX and to a lesser degree by AN-152. Estrogen receptors have a well-established role in bladder cancer progression [46, 47]. Similarly, androgen receptors are implicated in bladder carcinogenesis [48, 49]. The other hormonal receptors investigated, PPARs (peroxisome proliferator-activated receptors), RARs (retinoic acid receptors) and RXRs (retinoid X receptors) can regulate several processes important in tumor development, including cell proliferation, differentiation and apoptosis.

Transcription factors are involved in many processes, such as growth, differentiation, tumorigenesis and apoptosis. Treatment with DOX increased the expression of many genes related to transcription factors, especially in HT-1197 tumors. Since Karashima et al [50] verified that NFkBs (nuclear factor kappB) have a role in angiogenesis and metastasis of bladder cancers, we selected NFkBs and showed that NFkB was strongly increased by DOX but not by AN-152.

Pro- and anti-apoptotic genes in the tumors were affected differently by chronic administration of the two drugs. DOX changed pro-apoptotic genes, more favorably particularly in RT-4 cancers. DOX also strongly increased the expression of many anti-apoptotic genes; AN-152 caused fewer changes in these. Our short *in vitro* study revealed that both DOX and AN-152 significantly increased apoptosis in all three tumors. They acted similarly on J-82 cells and the apoptogenic action of AN-152 was much stronger than that of DOX on RT-4 and HT-1376 cancer cells.

The PCR arrays used in this study highlight essential differences between the action of AN-152 and DOX on various bladder cancers. The PCR arrays investigate a wide spectrum of tumor characteristics; our study analyzed over 150 genes. The pattern of changes, rather than individual changes in expression of single genes seemed to be more important. The functional pathways of groups of genes are interconnected. Thus, we separately investigated genes that affect cell cycle and transcription factors or those involved in DNA repair, but the combined effect determines whether a drug such as DOX will induce cell cycle arrest, repair, proliferation or apoptosis [51].

Summarizing the results of our study, we showed the expression of LH-RH receptors in tumors of 18 patients. We then demonstrated that cytotoxic LH-RH analog, AN-152, powerfully inhibits the growth of HT-1376, J82, RT-4 and HT-1197 human urinary bladder cancer lines xenografted into nude mice. All four lines express high affinity binding sites for LH-RH. The effect of AN-152 is stronger than that of DOX. Treatment with DOX activated strong multidrug resistance mechanisms in RT-4 and HT-1197 cancers, while AN-152 had little or no such effect. Based on our results, we suggest using these LHRH receptors for targeted cytotoxic treatment of bladder cancers with AN-152 (AEZS-108).

## MATERIALS AND METHODS

### Ethics Statement

Investigation has been conducted in accordance with the ethical standards and according to the Declaration of Helsinki and according to national and international guidelines and has been approved by the authors' institutional review board.

### Detection of LH-RH receptors in human bladder cancer specimens

Surgically removed specimens of 18 human primary urothelial bladder carcinomas were fixed in 4% neutral buffered formalin and embedded in paraffin. Sections were mounted on silanated glass slides (Sigma), and dried. For antigen retrieval, slides were immersed in 10mM citrate buffer, pH 6.0, heated for 15 min and then cooled to room temperature (RT). For immunostaining, the sections were blocked in 3% H2O2 in methanol for 10 min followed by BSA in Tris-buffered saline containing 0.1% Tween-20, pH 7.4 (TBS-T) for 1 h, and then incubated with primary LH-RH receptor antibody (A. Menarini Diagnostics, Germany) for 1 h at RT. Sections were incubated with peroxidase-conjugated mouse specific goat IgG (1:300, Dako, Real Envision, HRP Mouse) for 30 min at RT with color development over 30 sec to 10 min in diaminobenzidine. LH-RH receptor expression in malignant cells was graded as absent (-; LH-RHR expression in 0% of cells), weak expression (+; LH-RHR expression in 1-25% of cells), distinct expression (++; LH-RHR expression in 26-75% of cells) and strong expression (+++; LH-RHR expression in 75-100% of cells). Human pituitary (anterior lobe) served as positive control. For negative control, staining was performed without primary LH-RHR antibody. Two observers (GK and DB) graded tissues independently. The study was approved by the hospital ethics and research committees.

### Materials

Cytotoxic LH-RH analog, AN-152 (AEZS-108), and LH-RH antagonist, Cetrorelix, first synthesized in our laboratory [16] were provided by AEterna/Zentaris (Frankfurt am Main, Germany). [D-Trp^6^]LH-RH was obtained from Bachem (Torrance, CA, USA). DOX and other chemicals were purchased from Sigma (St. Louis, MO, USA). For treatment, the cytotoxic compounds were dissolved in 0.01% acetic acid, diluted with 5% mannitol and injected at 0.2 ml/20 g body weight.

### Animals and tumors

Female athymic nude mice (Ncr nu/nu) were from Charles River Laboratories International, Inc. (Durham, NC, USA) and Harlan Laboratories (Tampa, FL, USA). HT-1376, J82, RT-4 and HT-1197 tumor cells were from American Type Culture Collection (ATCC; Manassas, VA, USA). In vivo studies were performed using a subcutaneous xenograft model, which was induced as reported previously [52]. Briefly, six million cells were injected sc. into donor animals and 2 mm3 pieces of grown tumors were transplanted sc. into both flanks areas of experimental animals. The mice with grown tumors were randomly divided into groups of 7-8 mice each and treatment started. At experiments completion the mice were sacrificed, tumors and organs weighed and frozen.

### Experimental protocol

#### Experiment 1

HT-1376 tumors were transplanted sc. into 45 mice; treatment was started 85 days later (day 1). The groups were: 1) Control, 5% mannitol iv on days 1, 12, 20, 27, 34; 2) Cetrorelix, (depot preparation), 3 mg/mouse sc. on days 1, 22 and 43; 3) D-Trp6LH-RH, 25 μg/day/mouse sc daily; 4) AN-152 6.9 μmol/kg iv. on days 1, 12, 20, 27, 34; 5) DOX, 6.9 μmol/kg iv. on days 1, 12, 20, 27, 34. The experiment ended on day 61. The same doses of AN-152 and DOX were used in experiment 2, 3, and 4.

#### Experiment 2

J82 cancers were xenografted sc. into 36 mice. The treatment started 47 days after transplantation as follows: Group 1: Control; Group 2: AN-152 and Group 3: DOX, both given iv on days 1, 8, 15, 22, 29, 36 and 43. Experiment terminated at 136 days.

#### Experiment 3

RT-4 tumors were transplanted sc. into 32 mice; treatment started 49 days later (day 1). The groups: 1. Control; 2. AN-152; 3. DOX. Cytotoxic agents were administered iv. once weekly for 7 weeks. The controls received vehicle iv. Experiment terminated at 100 days.

#### Experiment 4

HT-1197 cancers were transplanted sc. into 40 mice. The treatment started 98 days later (day 1). The groups were: 1. Control, 2. AN-152; 3. DOX. The compounds were injected iv. on days 1, 5, 8, 12, 15, and AN-152 once more on day 30. Mice were sacrificed at 66 days.

### Receptor binding assays

Binding characteristics of receptors for LH-RH were determined by analyzing the binding of 125I-labeled [D-Trp^6^]LH-RH to tumor membrane homogenates from control mice as described [20].

### Molecular analysis

Total RNA was isolated from homogenized tumor samples from each group using the NucleoSpin kit (Macherey-Nagel, Bethlehem, PA, USA). Three samples per group were analyzed. Quality control of RNA samples was done as previously reported [53]. The Human Cancer Drug Resistance & Metabolism and the Human Apoptosis RT Profiler PCR Arrays (Qiagen, Inc. Valencia, CA, USA) were used to analyze mRNA levels of genes related to drug metabolism and apoptosis in tumors. Synthesis of cDNA, and real-time RT-PCR arrays were performed as described [54, 55]. Fold-changes in gene expression were calculated using the ΔΔCt method. Five housekeeping genes were used for normalization of the results.

For Western blots, bladder tumor tissue was processed as described [56]. Briefly, isolated proteins were sonicated and the lysates adjusted to equal concentrations. Primary antibodies for LH-RH receptors were purchased from Abcam (ab 58561 Cambridge, MA, USA), and for Bcl2 and BclX from Cell Signaling #2876 (Danvers, MA, USA) and Santa Cruz sc-8392 (Santa Cruz, CA, USA), respectively. The immunoreactive bands were visualized with the Odyssey Infrared Imaging System, using 3.0 software (LI-COR Biosciences, Lincoln, NE, USA).

### *In vitro* analysis of multi-drug resistance (MDR)

HT-1376, J82, and RT4 cells (105 per well) were seeded onto 96-well plates, cultured for 48 hours, and then treated for 4 hours with AN-152, [D-Trp^6^]LH-RH, DOX or the combination of [D-Trp^6^]LH-RH and DOX at a concentration of 1μM. As positive controls, cyclosporine A and verapamil were used (1:1000). Drug resistance was evaluated by using the Multi-Drug Resistance Assay Kit (Calcein AM) (Cayman Chemicals, Ann Arbor, MI). Fluorescence was measured in a Victor 3 Multilabel Counter (Perkin-Elmer, Waltham, MD) with excitation and emission wavelengths of 485 nm and 535 nm, respectively.

### *In vitro* analysis of apoptosis

HT-1376, J82, and RT4 cells (105 per well) were seeded onto 96-well plates, cultured for 48 hours, and then treated for 1 hour with AN-152, [D-Trp^6^]LH-RH, DOX or the combination of [D-Trp^6^]LH-RH and DOX at a concentration of 1μM. Apoptosis was detected using the Multi-Parameter Apoptosis Assay Kit (Cayman Chemicals). Fluorescence was measured in a Victor 3 Multilabel Counter with excitation and emission wavelengths of 560 nm and 595 nm, respectively to detect the fluorescence of TMRE (tetramethylrhodamine, ethyl ester) staining, and with excitation and emission wavelengths of 485 nm and 535 nm, respectively to detect early stage apoptotic cells stained by Annexin V FITC (fluorescein isothiocyanate).

### Statistical analysis

Sigmaplot 11.0 program (Systat Software, Inc. SigmaPlot for Windows; San Jose, CA, USA) was used for statistical evaluation. After analysis of variance, the groups were compared with Dunnett's method or with the Mann-Whitney Rank Sum Test and significance was accepted at P < 0.05.

## Supplementary Figures and Tables


