# Supplementary material for: Receptor-targeted therapy of human experimental urinary bladder cancers with cytotoxic LH-RH analog AN-152 (AEZS-108)
Source: Oncotarget. 2012 Jul 22;3(7):686–99. doi: 10.18632/oncotarget.546 (PMC3443252; doi:10.18632/oncotarget.546)
Supplement: Supplementary file 1 [file oncotarget-03-686-s001.pdf]

Fig. S1. Samples of human urinary bladder cancers grown in nude mice were analyzed with the Human Apoptosis RT Profiler PCR Array. The vertical bars on the left show 10-fold change compared to control. *=P<0.05 vs. control.

a) Changes in expression of anti-apoptotic genes. **AKT1**: V-akty murine thymoma viral oncogene homolog1; **BAG1**, **BAG3**, **BAG4**: BCL2 associated anthanogene1, 3, 4; **BCL2**: B-cell CLL/lymphoma 2; **BCL2A1**: BCL2-related protein A1; **BCL2L1**, **BCL2L10**, **BCL2L2**: BCL2-like 1, 10, 2; **BFAR**: Bifunctional apoptosis regulator; **NAIP**: NLR family, apoptosis inhibitory protein; **BIRC2**, **BIRC3**: Baculoviral IAP repeat containing 2, 3; **XIAP**: X-linked inhibitor of apoptosis; *15*: **BIRC6**, **BIRC8**: Baculovirus IAP repeat containing 6, 8; **BNIP1**, **BNIP2**: BCL2/adenovirus E1B 19kDa interacting protein 1, 2; **CD40LG**: CD40 molecule, TNF receptor superfamily member 5 ligand; **CFLAR**: CASP8 and FADD-like apoptosis regulator; **IGF1R**: Insulin-like growth factor 1 receptor; **MCL1:** Myeloid cell leukemia sequence 1; **NOL3**: Nucleolar protein 3; **TNFRSF11B**: Tumor necrosis factor receptor superfamily, member 11B; **TRAF2**, **TRAF4**: TNF receptor-associated factor 2, 4.

b) Changes in expression of pro-apoptotic genes. **ABL1**: C-abl oncogene 1, non-receptor tyrosine kinase; **APAF1**Apoptotic peptidase activating factor 1; **BAD**: BCL2-associated agonist of cell death; **BAK1**: BCL2-antagonist/killer 1; **BAX:** BCL2-associated X protein; **BCL10**: B-cell CLL/lymphoma 10; **BCL2L11**: BCL-like 11; **BCLAF1**: BCL2-associated transcription factor 1; **BID**: BH3 interacting domain death agonist; **BIK**: BCL2-interacting killer; **BNIP3**, **BNIP3L**: BCL2/adenovirus E1B 19kDa interacting protein 3, 3-like: **BRAF**: V-raf murine sarcoma viral oncogene homolog B1 *14*: **NOD1**: Nucleotide-binding oligomerization domain containing 1; **CARD6**, **CARD8**: Caspase recruitment domain family, member 6, 8; **CASP1**, **CASP10**, **CASP14**, **CASP2**, **CASP3**, **CASP4**, **CASP5**, **CASP6**, **CASP7**, **CASP8**, **CASP9**: Caspases 1-14; **CD40**: CD40 molecule, TNF receptor superfamily member 5; **CIDEA**, **CIDEB**: Cell death-inducing DFFA-like effector a, b; **CRADD**: CASP2 and RIPK1 domain containing adaptor with death domain; **DAPK1**Death-associated protein kinase 1; **DFFA**: DNA fragmentation factor, 45kDa. Alphas polypeptide; **FADD**: Fas (TNFRSFF6)-associated via death domain; **FAS**: Fas (TNF receptor superfamily, member 6; **FASLG**: Fas ligand; **GADD45A**Growth arrest and DNA-damage-inducible **HRK**: Harakiri, BCL2 interacting protein; **LTA**, **LTBR**: Lymphotoxin alpha, beta receptor; **PYCARD**PYD and CARD domain containing; **RIPK2**: Receptor-interacting serine-threonine kinase 2; **TNF**: Tumor necrosis factor; **TNFRSF10A**, **TNFRSF10B**, **TNFRSF1A**, **TNFRSF21**, **TNFRSF25**: Tumor necrosis factor receptor superfamily, members 10a, 10b, 1a, 21, 25; **CD27**: CD27 molecule; **TNFRSF9**: Tumor necrosis factor receptor superfamily, member 9; **TNFSF10**: Tumor necrosis factor (ligand) superfamily, member 10; **CD70**: CD70 molecule; **TNFSF8**: Tumor necrosis factor (ligand) superfamily, member 8; **TP53**: Tumor protein p53; **TP53BP2**Tumor protein p53 binding protein, 2; **TP73**: Tumor protein p73; **TRADD**: TNFRSF1A-associated via death domain; **TRAF3:** TNF receptor-associated factor 3.

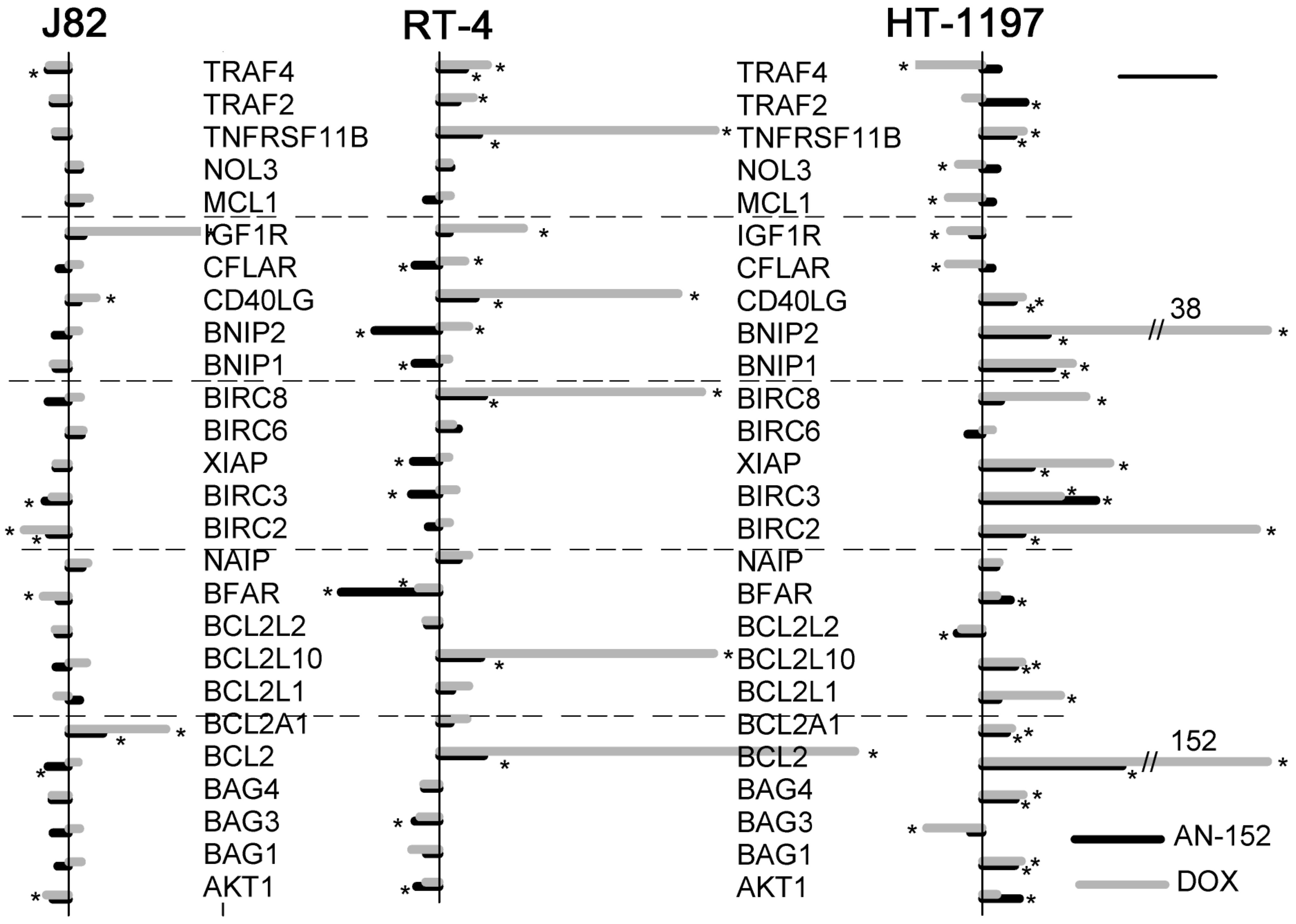
J82
RT-4
HT-1197
TRAF4
TRAF2
TNFRSF11B
NOL3
MCL1
IGF1R
CFLAR
CD40LG
BNIP2
BNIP1
BIRC8
BIRC6
XIAP
BIRC3
BIRC2
NAIP
BFAR
BCL2L2
BCL2L10
BCL2L1
BCL2A1
BCL2
BAG4
BAG3
BAG1
AKT1
38
152
AN-152
DOX

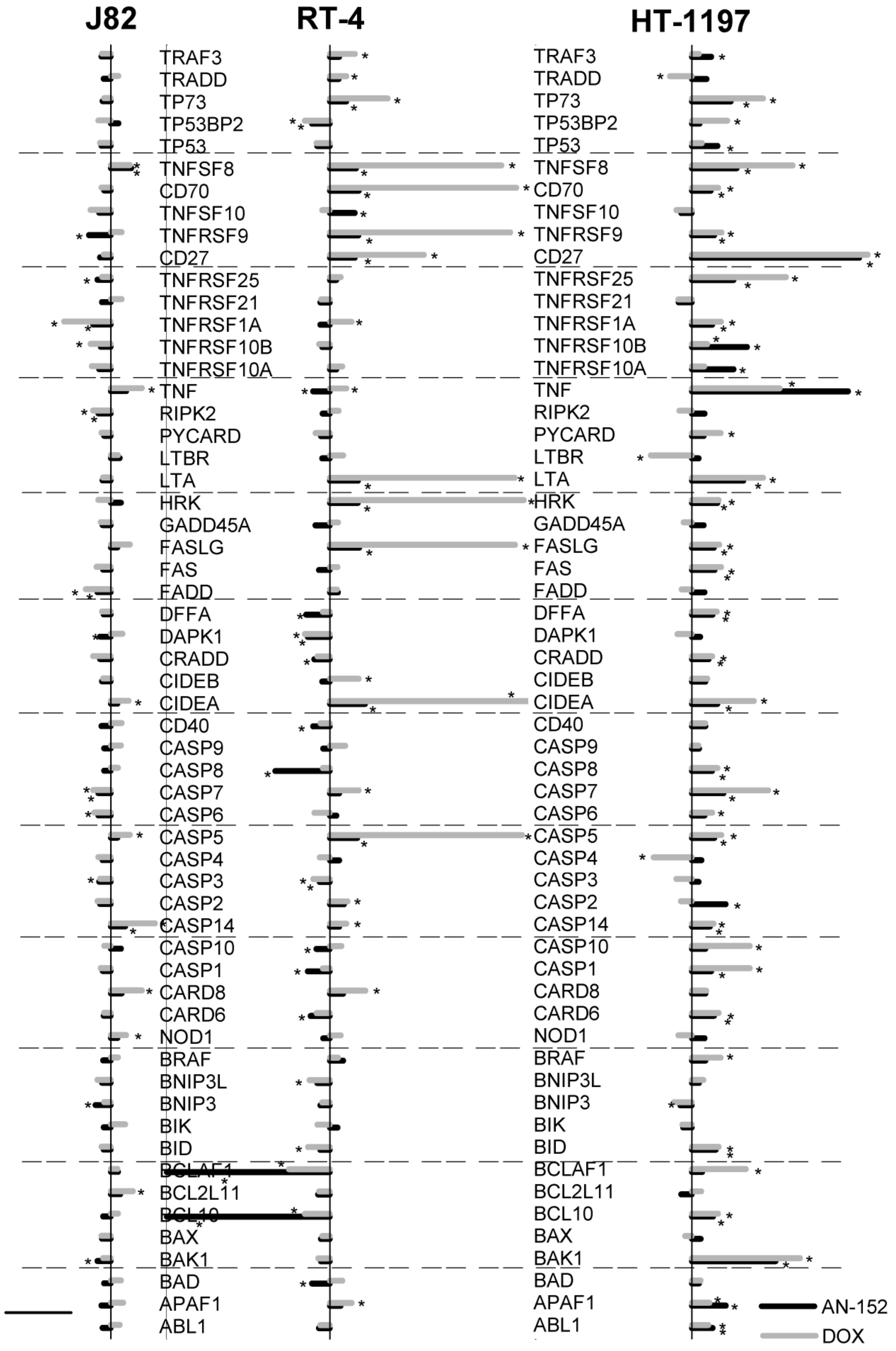
J82
RT-4
HT-1197
TRAF3
TRADD
TP73
TP53BP2
TP53
TNFSF8
CD70
TNFSF10
TNFRSF9
CD27
TNFRSF25
TNFRSF21
TNFRSF1A
TNFRSF10B
TNFRSF10A
TNF
RIPK2
PYCARD
LTBR
LTA
HRK
GADD45A
FASLG
FAS
FADD
DFFA
DAPK1
CRADD
CIDEB
CIDEA
CD40
CASP9
CASP8
CASP7
CASP6
CASP5
CASP4
CASP3
CASP2
CASP14
CASP10
CASP1
CARD8
CARD6
NOD1
BRAF
BNIP3L
BNIP3
BIK
BID
BCLAF1
BCL2L11
BCL10
BAX
BAK1
BAD
APAF1
ABL1
AN-152
DOX

Supplemental Table

Table S1. Binding characteristics of LH-RH receptors in human urinary bladder cancers grown in nude mice

Tumor	B_{max} (fmol/mg membrane protein)	K_d (nM)
HT-1376	658.4 ± 80.1	8.36 ± 0.41
J82	330.8 ± 35.5	4.24 ± 0.12
RT-4	260.8 ± 12.3	4.65 ± 0.09
HT-1197	500.5 ± 12.4	8.55 ± 0.37

The values are means ± SE. Two-three binding experiments were done in duplicate or triplicate. B_{max}: maximal binding capacity; K_d: dissociation constant.
